# 32 Food Security as a Predictor of Global Pediatric Postburn Mortality

**DOI:** 10.1093/jbcr/irad045.006

**Published:** 2023-05-15

**Authors:** Rafael Felix Tiongco, Ayman Ali, Joseph Puthumana, C Scott Hultman, Julie Caffrey, Richard Redett, Carisa Cooney

**Affiliations:** Johns Hopkins University School of Medicine, Baltimore, Maryland; Duke University School of Medicine, Durham, North Carolina; Johns Hopkins University School of Medicine, Baltimore, Maryland; Johns Hopkins University School of Medicine, Baltimore, Maryland; Johns Hopkins University School of Medicine, Baltimore, Maryland; Johns Hopkins University School of Medicine, Baltimore, Maryland; Johns Hopkins University School of Medicine, Baltimore, Maryland; Johns Hopkins University School of Medicine, Baltimore, Maryland; Duke University School of Medicine, Durham, North Carolina; Johns Hopkins University School of Medicine, Baltimore, Maryland; Johns Hopkins University School of Medicine, Baltimore, Maryland; Johns Hopkins University School of Medicine, Baltimore, Maryland; Johns Hopkins University School of Medicine, Baltimore, Maryland; Johns Hopkins University School of Medicine, Baltimore, Maryland; Johns Hopkins University School of Medicine, Baltimore, Maryland; Duke University School of Medicine, Durham, North Carolina; Johns Hopkins University School of Medicine, Baltimore, Maryland; Johns Hopkins University School of Medicine, Baltimore, Maryland; Johns Hopkins University School of Medicine, Baltimore, Maryland; Johns Hopkins University School of Medicine, Baltimore, Maryland; Johns Hopkins University School of Medicine, Baltimore, Maryland; Johns Hopkins University School of Medicine, Baltimore, Maryland; Duke University School of Medicine, Durham, North Carolina; Johns Hopkins University School of Medicine, Baltimore, Maryland; Johns Hopkins University School of Medicine, Baltimore, Maryland; Johns Hopkins University School of Medicine, Baltimore, Maryland; Johns Hopkins University School of Medicine, Baltimore, Maryland; Johns Hopkins University School of Medicine, Baltimore, Maryland; Johns Hopkins University School of Medicine, Baltimore, Maryland; Duke University School of Medicine, Durham, North Carolina; Johns Hopkins University School of Medicine, Baltimore, Maryland; Johns Hopkins University School of Medicine, Baltimore, Maryland; Johns Hopkins University School of Medicine, Baltimore, Maryland; Johns Hopkins University School of Medicine, Baltimore, Maryland; Johns Hopkins University School of Medicine, Baltimore, Maryland; Johns Hopkins University School of Medicine, Baltimore, Maryland; Duke University School of Medicine, Durham, North Carolina; Johns Hopkins University School of Medicine, Baltimore, Maryland; Johns Hopkins University School of Medicine, Baltimore, Maryland; Johns Hopkins University School of Medicine, Baltimore, Maryland; Johns Hopkins University School of Medicine, Baltimore, Maryland; Johns Hopkins University School of Medicine, Baltimore, Maryland; Johns Hopkins University School of Medicine, Baltimore, Maryland; Duke University School of Medicine, Durham, North Carolina; Johns Hopkins University School of Medicine, Baltimore, Maryland; Johns Hopkins University School of Medicine, Baltimore, Maryland; Johns Hopkins University School of Medicine, Baltimore, Maryland; Johns Hopkins University School of Medicine, Baltimore, Maryland; Johns Hopkins University School of Medicine, Baltimore, Maryland

## Abstract

**Introduction:**

Food security (FS) is defined as access to sufficient and nutritious food. Children are disproportionately affected by low FS. Globally, burns contribute to over 50% of the disability-adjusted life years lost among 0-14 year-old children. Given the significance of nutrition in postburn outcomes, we hypothesized that high FS would be predictive of decreased global postburn mortality in pediatric patients.

**Methods:**

Publicly-available, deidentified datasets were obtained from the World Health Organization’s Global Burn Registry (GBR) and Corteva Agriculture’s Global FS Index (GFSI). The GFSI calculates a FS score annually from intergovernmental organization data reviewed by a panel of experts. FS score was reported on a scale from 0-100 with 100 indicating the highest FS. The GFSI also groups countries into “Good” (FS score ≥ 60) or “Moderate” (FS Score ≥ 40) FS environments (FSEs). Patients aged 0-19 years were included; after linking GBR and GFSI datasets, countries with < 100 patients were excluded. Descriptive statistics and non-parametric tests were performed on data stratified by FSE. Multiple logistic regression controlling for %TBSA was used to predict mortality with FS score. All statistical analyses were conducted in R version 4.1.2.

**Results:**

From 2016-2020, there were 2,246 cases over nine countries: Peru (n=839, 37.4%), Tanzania (n=290, 12.9%), India (n=225, 10.0%), Nigeria (n=195, 8.7%), Pakistan (n=181, 8.1%), Kenya (n=166, 7.4%), Nepal (n=145, 6.5%), South Africa (n=105, 4.7%), and Mexico (n=100, 4.5%). Median age was 3.0 [IQR 2.0, 7.0] years with 42.7% female. Median %TBSA was 15.0 [IQR 5.0, 25.0]%. Most injuries were by hot liquid, steam, or gas (n=1,457, 64.9%) followed by flame (n=560, 24.9%). There were 259 deaths (11.5%).

Stratified by FSE, 939 cases occurred in “Good” and 1,307 in “Moderate” FSE countries. More flame injuries occurred in Moderate (n=373, 28.5%) vs. Good (187, 19.9%, p< 0.001) FSEs while more hot liquid, steam, or gas injuries occurred in Good (n=688, 73.3%) vs. Moderate (n=769, 58.8%, p< 0.001) FSEs. Mortality was higher in Moderate (n=245, 18.7%) vs. Good (n=14, 1.5%, p< 0.001) FSEs.

Regression analysis controlling for %TBSA showed increased FS score was associated with decreased odds of pediatric postburn mortality [multivariable OR 0.77 (95%CI 0.72-0.81), p< 0.001]. Additionally, urban vs. rural patient residence was associated with decreased odds of postburn mortality [multivariable OR 0.36 (95%CI 0.16-0.85), p=0.015].

**Conclusions:**

Our study shows that increasing FS score was associated with decreased odds of pediatric postburn mortality. Additionally, mortality and flame injuries were higher in Moderate vs. Good FSE-grouped countries. International efforts to increase FS may improve survival in pediatric burn patients.

**Applicability of Research to Practice:**

Improving food security is an actionable item that may improve pediatric postburn outcomes regardless of the country’s FSE-grouping.

